# Immune-Related Neurological Toxicities of PD-1/PD-L1 Inhibitors in Cancer Patients: A Systematic Review and Meta-Analysis

**DOI:** 10.3389/fimmu.2020.595655

**Published:** 2020-12-18

**Authors:** Yuan Tian, Aiqin Gao, Qing Wen, Shuyun Wang, Shuisheng Zhang, Xiaowei Yang, Guohai Su, Yuping Sun

**Affiliations:** ^1^ Department of Oncology, Jinan Central Hospital Affiliated to Shandong University, Jinan, China; ^2^ Department of Radiotherapy Oncology, Shandong Provincial Qianfoshan Hospital, Shandong University, Jinan, China; ^3^ Department of Radiotherapy Oncology, Shandong Provincial Qianfoshan Hospital, The First Hospital Affiliated With Shandong First Medical University, Jinan, China; ^4^ Jinan Clinical Research Center of Shandong First Medical University, Jinan, China; ^5^ Department of General Surgery, Peking University Third Hospital, Beijing, China; ^6^ Department of Hepatobiliary Intervention, Beijing Tsinghua Changgung Hospital, School of Clinical Medicine, Tsinghua University, Beijing, China; ^7^ Department of Cardiovascular Diseases, Jinan Central Hospital Affiliated to Shandong University, Jinan, China; ^8^ Department of Oncology, Jinan Central Hospital affiliated to Shandong First Medical University, Jinan, China

**Keywords:** neurological toxicities, cancer, meta-analysis, PD-1, PD-L1

## Abstract

**Background:**

Systematic assessment of PD-1/PD-L1 inhibitor-related neurological toxicities is important for guiding anti-PD-1 and anti-PD-L1 immunotherapy. Therefore, we conducted this meta-analysis to reveal the relationship between PD-1/PD-L1 inhibitors and neurological toxicities among cancer patients.

**Methods:**

Clinical trials investigating PD-1/PD-L1 inhibitors in cancer patients were identified by a systematic search of PubMed. The random-effect model was used to synthesize individual studies. Neurological toxicities, including all-grades and grades 3–5, were taken into account for the final comprehensive meta-analysis. The Newcastle Ottawa Scale (NOS) was used to assess the quality of included trials.

**Results:**

Thirty-one clinical trials containing data of neurological toxicities were included. Compared with chemotherapy, the risk of all-grade neurological toxicities caused by PD-1/PD-L1 inhibitors was much lower in terms of peripheral neuropathy [OR = 0.07, 95%CI:(0.04, 0.13)], peripheral sensory neuropathy [OR = 0.07, 95%CI(0.04, 0.12)], dysgeusia [OR = 0.26, 95%CI:(0.19, 0.35)], paraesthesia [OR = 0.23, 95%CI:(0.14, 0.36)], and polyneuropathy [OR = 0.12, 95%CI:(0.01, 0.94)]. However, for grades 3–5, the statistically significant results were only seen in peripheral neuropathy [OR = 0.15, 95%CI:(0.07, 0.34)] and peripheral sensory neuropathy [OR = 0.13, 95%CI:(0.04, 0.40)]. No statistically significant difference regarding the risk of headache, dizziness, and Guillain–Barré syndrome was found between PD-1/PD-L1 inhibitors and chemotherapy. For PD-1/PD-L1 inhibitors plus chemotherapy, the risk trends of the above-mentioned neurological toxicities, especially grades 3–5 peripheral neuropathy [OR = 1.76, 95%CI:(1.10, 2.82)] was increased compared to chemotherapy alone.

**Conclusion:**

Our comprehensive analysis showed that PD-1/PD-L1 inhibitors alone exhibited lower neurological toxicities than chemotherapy. However, the risk of headache, dizziness, and Guillain–Barré syndrome was similar between PD-1/PD-L1 and chemotherapy. For PD-1/PD-L1 inhibitors plus chemotherapy, the incidence trend of neurological toxicities would be increased, especially for peripheral neuropathy of grades 3–5.

## Introduction

Cancer immunotherapies, developed to overcome the immune escape mechanisms of cancer progression and metastatic dissemination, are becoming familiar to oncologists (1), especially for programmed cell death protein 1 (PD-1) and its ligand (PD-L1) inhibitors. PD-1/PD-L1 inhibitors belong to immune checkpoint blocking drugs (1); they can block the binding of tumor cells to PD-1 of T cells by means of PD-L1, restore the ability to recognize tumor cells, and further restore the cell recognition and killing ability of T cells (1). Immunotherapies, including cytotoxic T lymphocyte antigen-4 (CTLA-4) and PD-1/PD-L1 had changed the treatment landscape for plenty of solid tumors but conferred unique toxicity profiles owing to their unique mechanism of actions (1–3).

Most of those toxic reactions had aroused sufficient attention from clinicians and researchers, and guidelines for related treatment had been developed for reference (2, 4). Neurological toxicities, including peripheral neuropathy, peripheral sensory neuropathy, peripheral motor neuropathy, dysgeusia, paraesthesia, headache, dizziness, Guillain–Barré syndrome, neurotoxicity, myasthenia gravis, noninfectious encephalitis/myelitis, and polyneuropathy, were mostly reported in the form of case reports or reviews and were considered to be rare immune-related adverse events (1, 5–14). The appearance of neurological toxicities might be diverse, involving any aspect of the central or peripheral nervous system accompanied by different diagnostic signs and symptoms (1).

As more and more clinical trials investigating the clinical efficacy and safety of PD-1/PD-L1 in cancer patients are being conducted, various treatment induced adverse events had been gradually reported (1, 2). However, regarding the neurological toxicities of PD-1/PD-L1, no systematic reviews and meta-analysis have been conducted in this regard (1–14). Therefore, in order to clarify the relationship between PD-1/PD-L1 inhibitors and the risk of neurological toxicities, this systematic review and meta-analysis was conducted.

## Method

This research was conducted and reported according to the guidelines of the Preferred Reporting Items for Systematic Reviews and Meta-analyses (PRISMA) (15).

### Types of Enrolled Studies

Randomized, open-label, controlled clinical trials investigating the efficacy and safety of PD-1/PD-L1 inhibitors in cancer patients were included. Phase III clinical trials, limited to solid tumors, were given a priority. Then, clinical trials of other phases would be checked for eligibility and placed in an alternative location. Clinical trials investigating hematological malignancies were beyond our consideration. In order to collect as many articles as possible, the control group was not restricted to a certain therapeutic agent or intervention. For inclusion, the study must report the data of at least one type of neurological toxicities related to immunotherapy. Articles must be published in English.

### Search Strategy

Keywords, including neoplasm, cancer, precancer, malignant, premalignant, tumor, PD-1, PD-L1, and clinical trial, were used for the PubMed search with reference to participants, interventions, comparisons, outcomes, and study design (PICOS) (15). The published date was limited to the last 10 years (July 9, 2010 to July 9, 2020). Of note, some data regarding peripheral neuropathy was also collected from a former systematic review and meta-analysis (16). Four authors were designated to check the eligibility of all retrieved reports. They were also responsible for the extraction of relevant data from finally included trials. In the case of duplicated clinical trials, only one was included in the final analysis step. The corresponding authors (YS and GS) were responsible for resolving all disagreements.

### Evaluation of Study Quality and Publication Bias

Funnel plots, Egger’s test, and the Newcastle-Ottawa scale (NOS) were used to check publication bias and risk of bias of individual trials, respectively (15, 17–20). The quality assessment included the appraisal of random sequence generation, allocation concealment, blinding of participants and personnel, blinding of outcome assessment, incomplete outcome data, and selective outcome reporting (shown in a single figure). Harbord’s test was used to check the risk of publication bias of enrolled clinical trials (21). A *P-value* of <0.05 was used as the cut-off value for statistical significance.

### Outcome and Exposure of Interest

Any data of neurological toxicities, including peripheral neuropathy, peripheral sensory neuropathy, peripheral motor neuropathy, dysgeusia, paraesthesia, headache, dizziness, Guillain-Barré syndrome, neurotoxicity, and polyneuropathy, were collected and further analyzed. Baseline characteristics of included articles are summarized in (**Table 1**). The risk of neurological toxicities relating to all grades was our primary outcome of interest in the final meta-analysis. Grading of neurological toxicities ranged from one (mild symptoms that do not interfere with activities of daily living) to five (fatal neurological toxicities).

**Table 1:** Baseline characteristics of included studies (N = 37 articles of 31 clinical trials).

NO	Reference	NCT Number	Trial Name	Drug Name	PD-1/PD-L1	Treatment Regimen	Previous Therapy	Phase	Tumor Type	Involving Patients
1	Motzer et al. (22)	NCT02684006	JAVELIN Renal 101	Avelumab	PD-L1	Avelumab + Axitinib vs. Sunitinib	NO	III	RCC	873
2	Rini et al. (23)	NCT02420821	IMmotion151	Atezolizumab	PD-L1	Atezolizumab + Bevacizumab vs. Sunitinib	NO	III	RCC	897
3	Mok et al. (24)	NCT02220894	KEYNOTE-042	Pembrolizumab	PD-1	Pembrolizumab vs. Platinum-based Chemotherapy	NO	III	NSCLC	1241
4	Cohen et al. (25)	NCT02252042	KEYNOTE-040	Pembrolizumab	PD-1	Pembrolizumab vs. (Methotrexate, Docetaxel, Cetuximab)	YES	III	HNSCC	480
5	Schmid et al. (26)	NCT02425891	IMpassion130	Atezolizumab	PD-L1	Atezolizumab + Nab-paclitaxel vs. Nab-paclitaxel	NO	III	BC	890
6	Horn et al. (27)	NCT02763579	IMpower133	Atezolizumab	PD-L1	Atezolizumab + CE vs. CE	NO	III	SCLC	394
7	Socinski et al. (28)	NCT02366143	IMpower150	Atezolizumab	PD-L1	Atezolizumab + BCP vs. BCP	NO	III	NSCLC	787
8	Paz-Ares et al. (29)	NCT02775435	KEYNOTE-407	Pembrolizumab	PD-1	Pembrolizumab + CP vs. CP	NO	III	NSCLC	558
9	Barlesi et al. (30)	NCT02395172	JAVELIN Lung 200	Avelumab	PD-L1	Avelumab vs. Docetaxel	YES	III	NSCLC	792
10	Shitara et al. (31)	NCT02370498	KEYNOTE-061	Pembrolizumab	PD-1	Pembrolizumab vs. Paclitaxel	YES	III	Gastric or junction Cancer	570
11	Powles et al. (32)	NCT02302807	IMvigor211	Atezolizumab	PD-L1	Atezolizumab vs. Vinflunine, Paclitaxel, or Docetaxel	YES	III	UC	902
12	Hida et al. (33)	NCT02008227	OAK	Atezolizumab	PD-L1	Atezolizumab vs. Docetaxel	YES	III	NSCLC	101
13	Bellmunt et al. (34)	NCT02256436	KEYNOTE-045	Pembrolizumab	PD-1	Pembrolizumab vs. Paclitaxel, Docetaxel, or Vinflunine	YES	III	UC	521
14	Rittmeyer et al. (35)	NCT02008227	OAK	Atezolizumab	PD-L1	Atezolizumab vs. Docetaxel	YES	III	NSCLC	1187
15	Langer et al. (36)	NCT02039674	KEYNOTE-021	Pembrolizumab	PD-1	Pembrolizumab + PC vs. PC	NO	II	NSCLC	121
16	Reck et al. (37)	NCT02142738	KEYNOTE-024	Pembrolizumab	PD-1	Pembrolizumab vs. Platinum-based chemotherapy	NO	III	NSCLC	304
17	Ferris et al. (38)	NCT02105636	CheckMate 141	Nivolumab	PD-1	Nivolumab vs. (Methotrexate, Docetaxel, or Cetuximab)	YES	III	HNSCC	347
18	Antonia et al. (39)	NCT01928394	CheckMate 032	Nivolumab	PD-1	Nivolumab vs. Nivolumab + Ipilimumab	YES	I/II	SCLC	213
19	Fehrenbacher et al. (40)	NCT01903993	POPLAR	Atezolizumab	PD-L1	Atezolizumab vs. Docetaxel	YES	II	NSCLC	277
20	Herbst et al. (41)	NCT01905657	KEYNOTE-010	Pembrolizumab	PD-1	Pembrolizumab vs. Docetaxel	YES	II/III	NSCLC	991
21	Hodi et al. (42)	NCT01927419	CheckMate 069	Nivolumab	PD-1	Nivolumab + Ipilimumab vs. Ipilimumab	NO	III	Melanoma	140
22	Borghaei et al. (43)	NCT01673867	CheckMate 057	Nivolumab	PD-1	Nivolumab vs. Docetaxel	YES	III	NSCLC	555
23	Brahmer et al. (44)	NCT01642004	CheckMate 017	Nivolumab	PD-1	Nivolumab vs. Docetaxel	YES	III	NSCLC	260
24	Motzer et al. (45)	NCT01668784	CheckMate 025	Nivolumab	PD-1	Nivolumab vs. Everolimus	YES	III	RCC	821
25	Kato et al. (46)	NCT02569242	ATTRACTION-3	Nivolumab	PD-1	Nivolumab vs. Paclitaxel or Docetaxel	YES	III	OSCC	417
26	Gandhi et al. (47)	NCT02578680	KEYNOTE-189	Pembrolizumab	PD-1	Pembrolizumab + PC vs. PC	NO	III	NSCLC	439
27	Ascierto et al. (48)	NCT02130466	N/A	Pembrolizumab	PD-1	Pembrolizumab + DT vs. DT	NO	II	Melanoma	120
28	Paz-Ares et al. (49)	NCT03043872	CASPIAN	Durvalumab	PD-L1	Durvalumab + EP vs. EP	NO	III	SCLC	431
29	Schmid et al. (50)	NCT03036488	KEYNOTE-522	Pembrolizumab	PD-1	Pembrolizumab + CP vs. CP	NO	III	TNBC	1170
30	Hodi et al. (51)	NCT01844505	CheckMate 067	Nivolumab	PD-1	Nivolumab +Iipilimumab or Nivolumab alone vs. Ipilimumab	NO	III	Melanoma	937
31	Wolchok et al. (52)									
32	Larkin et al. (53)									
33	Larkin et al. (54)									
34	Antonia et al. (55)	NCT02125461	PACIFIC	Durvalumab	PD-L1	Durvalumab vs. placebo	YES	III	NSCLC	709
35	Antonia et al. (56)									
36	Hui et al. (57)									

vs., Versus; N/A, Not Available; RCC, Renal Cell Carcinoma; NSCLC, Non Small Cell Lung Cancer; HNSCC, Head-and-Neck Squamous Cell Carcinoma; SCLC, Small Cell Lung Cancer; EC, Etoposide + Carboplatin; BCP, Bevacizumab plus Carboplatin plus Paclitaxel; CP, Carboplatin + Paclitaxel; UC, Urothelial Carcinoma; OSCC, Oesophageal Squamous Cell Carcinoma; DT, Dabrafenib + Trametinib; TNBC, Triple-Negative Breast Cancer; BC, Breast Cancer; UC, Urothelial Carcinoma.

### Assessment of Heterogeneity and Statistical Analysis

Heterogeneity of all enrolled clinical trials was identified by Cochrane’s Q statistic test (21). The grade of heterogeneity was estimated by the DerSimonian–Laird method and I^2^ values together, which was suggested by Higgins and colleagues (15, 21). Heterogeneity was deemed to be low, moderate, or high according to I^2^ values < 25, 25–50, and > 50%, respectively (16). All data analyses were completed by the software Review Manager 5.3. Owing to the existence of inherent heterogeneity among included trials, the random effect (RE) was used for the evaluation of odds ratio (OR) and their corresponding 95% confidence interval (CI) (58). Sometimes, the fixed effects (FE) model was used as a supplement. All reported *P* values are two-sided, and *P*<0.05 was deemed to be statistically significant. Subgroup analysis was made according to tumor types, treatment regimens, and PD-1/PD-L1 inhibitors.

## Results

### Literature Search Results

**Figure 1:**
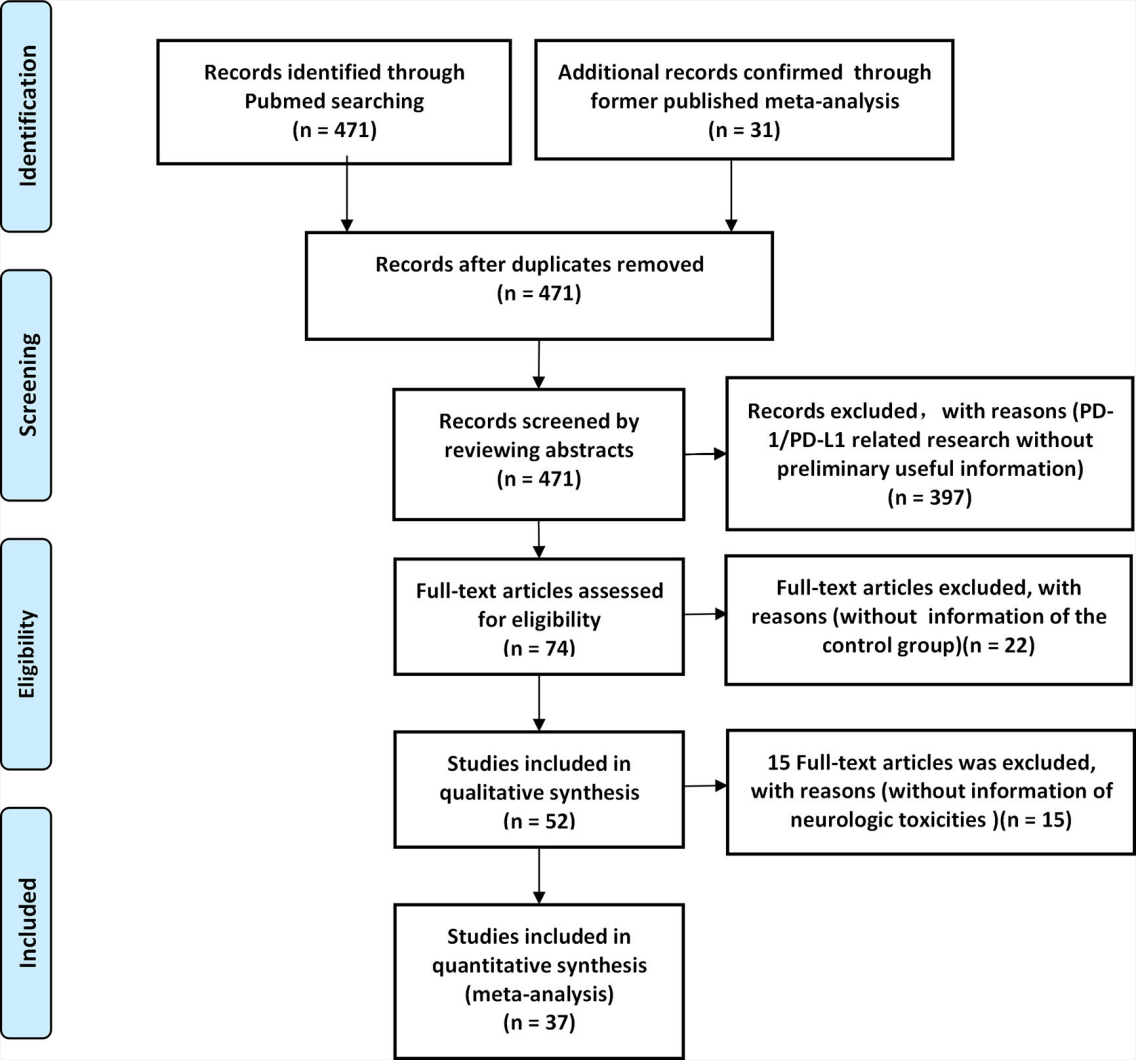
A PRISMA flow diagram of the screening process of our review.

**Figure 2:**
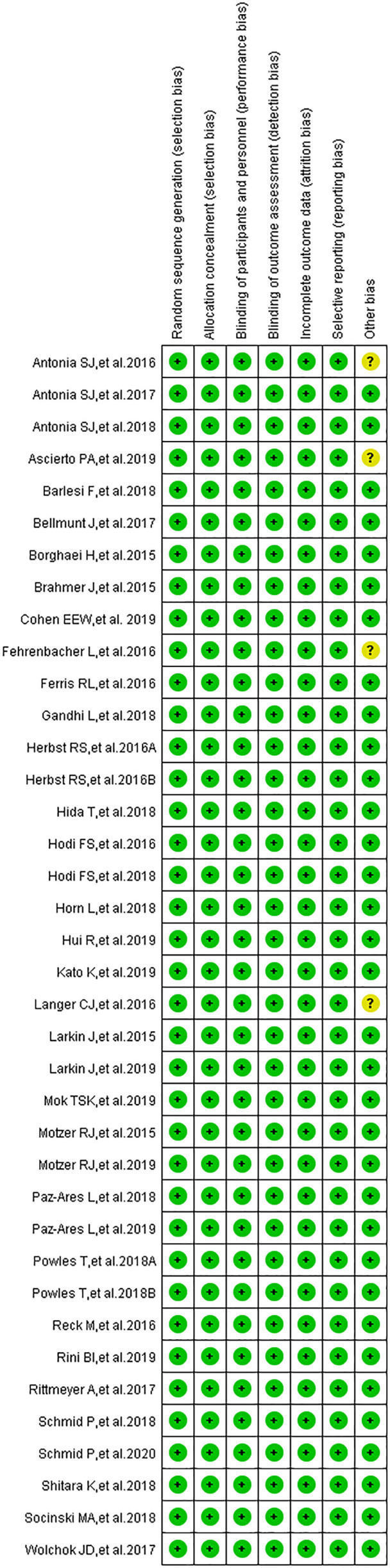
A summary of the quality (risk of bias) of included studies.

A total of 471 PD-1/PD-L1 inhibitor-related clinical trials were identified through PubMed, while 31 related studies were collected from the former published meta-analysis (16). Fifty-two articles met our preliminary screening criteria, of which 36 articles (reporting the data of neurological toxicities of 31 clinical trials involving 9960 patients) were included in the final analysis phase (22–57). Results of different periods of the same clinical trial ‘CheckMate 067’ (NCT01844505) were reported by four articles (51–54), while the results of the clinical trial ‘PACIFIC’ (NCT02125461) was reported by three articles (55–57). The baseline characteristics of the 36 enrolled articles are displayed in (**Table 1**) **(** 22–57). The PRISMA flow diagram of the screening process of our review was provided in (**Figure 1**), while the quality of included studies is shown in (**Figure 2**) (22–57). After reviewing the full-texts of all included trials, 10 types of neurological toxicities were reported, including peripheral neuropathy (24–32, 34, 35, 38–41, 43, 44, 46, 50), peripheral sensory neuropathy (24–26, 29–34, 41, 42, 46, 50), dysgeusia (22, 23, 25, 26, 32–37, 41–43, 45, 47, 50), paraesthesia (25, 28, 32, 41–44, 49), headache (22, 23, 25, 26, 34, 41, 43, 47, 48, 51–57), dizziness (22, 25, 34, 36, 38, 41–44, 47, 51, 52), peripheral motor neuropathy (51), Guillain–Barré syndrome (25, 27, 33, 42, 51), neurotoxicity (25), and polyneuropathy (10, 25, 51).

### Characteristics of Identified Trials

Twenty-five studies were phase III clinical trials (22–35, 37, 38, 47–49, 49–57), three were phase II trials (36, 40, 48), one was phase I/II trial (39), and one was phase II/III trial (41). Twelve clinical trials (reported in 14 articles) investigated PD-L1 (22, 23, 26–28, 30, 32, 33, 35, 40, 49, 55–57), while the remaining 18 clinical trials (reported in 22 articles) investigated PD-1 (24, 25, 29, 31, 34, 36–39, 41–48, 50–53). Among included clinical trials, nine types of tumors were reported, including non-small cell lung cancer (NSCLC) (N = 14) (24, 28–30, 33, 35–37, 40, 41, 43, 44, 47, 55–57), small cell lung cancer (SCLC) (N = 3) (27, 39, 49), renal cell carcinoma (RCC) (N = 3) (22, 23, 45), esophageal squamous cell carcinoma (OSCC) (N = 1) (46), head and neck squamous cell carcinoma (HNSCC) (N = 2) (25, 38), urothelial cancer (UC) (N = 2) (32, 34), breast cancer (BC) (N = 2) (26, 50), melanoma (N = 3) (42, 48, 51–53, 56), and gastric or junction cancer (N = 1) (31). Previous therapies were reported in 16 clinical trials (25, 30–35, 38–41, 43–46, 55–56), while PD-1/PD-L1 inhibitors were administered as a first-line therapy in the remaining 15 clinical trials (22–24, 26–29, 36, 37, 42, 47–54).

### Risk of Bias

The results of the publication bias assessment, in the form of funnel plots, are provided in the supplement (**Supplementary Figures 1**–**3**, **5**, **7**, **9**) (15, 17–20, 22–57). Low risk of bias was identified in all clinical trials regarding selection bias, performance bias, detection bias, attrition bias, and reporting bias (**Figure 2**) (22–57). An unclear risk relating to other biases was identified in four clinical trials (36, 39, 40, 48). None of the included trials had a high risk of bias.

### Risk of Peripheral Neuropathy

**Figure 3:**
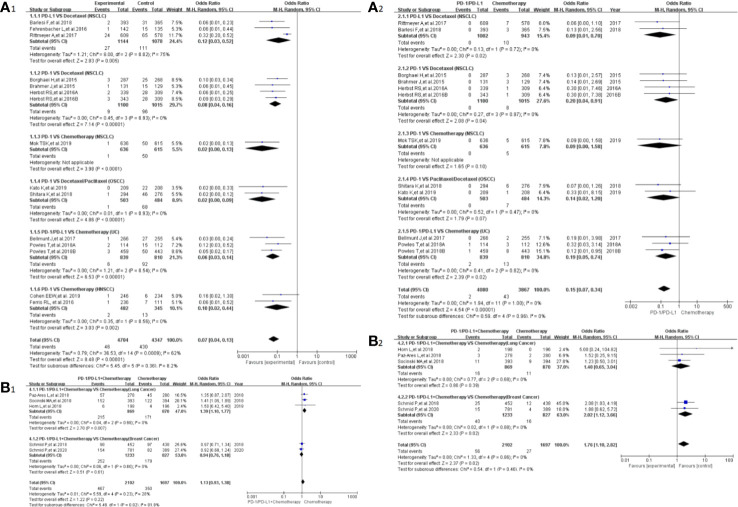
Forest plots of the risk of peripheral neuropathy. **(A1)** The risk of all-grade peripheral neuropathy calculated by the random effect (RE) model (PD-1/PD-L1 *vs* chemotherapy): subgroup analysis was put into practice based on PD-1/PD-L1 and tumor types in both groups. **(A2)** The risk of peripheral neuropathy of grades 3–5 calculated by the random effect (RE) model (PD-1/PD-L1 *vs* chemotherapy): subgroup analysis was put into practice based on PD-1/PD-L1 and tumor types in both groups. **(B1)** The risk of all grade peripheral neuropathy calculated by the random effect (RE) model (PD-1/PD-L1 + chemotherapy *vs* chemotherapy): subgroup analysis was put into practice based on tumor types in both groups. **(B2)** The risk of peripheral neuropathy of grades 3–5 calculated by the random effect (RE) model (PD-1/PD-L1 + chemotherapy *vs* chemotherapy): subgroup analysis was put into practice based on tumor types in both groups.

Peripheral neuropathy was reported in 20 clinical trials (24–32, 34, 35, 38–41, 43, 44, 46, 50), 19 of which were included in the final meta-analysis (24–32, 34, 35, 38, 40, 41, 43, 44, 46, 50). When PD-1/PD-L1 inhibitors were compared with chemotherapy, the risk of peripheral neuropathy of all grades was noticeably lower [OR = 0.07, 95%CI:(0.04, 0.13), I^2^ = 62%, Z = 8.48 (*P <* 0.00001); **Figure 3A1**], even for every subgroup relating to different tumor types (24–26, 30–32, 34, 38, 40, 41, 43, 44, 46). High heterogeneity was found (I^2^ = 62%), which was caused mainly by the NSCLC subgroup involving PD-L1 inhibitors (I^2^ = 75%, **Figure 3A1**) (26, 30, 40). The corresponding funnel plot is provided in the supplement (**S Figure 1A1**). Similarly, reduced risk of peripheral neuropathy of grades 3^–^5 was also noted [OR = 0.15, 95%CI:(0.07, 0.340, I^2^ = 0%, Z = 8.48 (*P <*0.00001); **Figure 3A2**]. The corresponding funnel plot is provided in the supplement (**S Figure 1A2**) (24, 26, 30–32, 34, 41, 43, 44, 46).

When PD-1/PD-L1 inhibitors plus chemotherapy were compared with chemotherapy (**Figures 3B1, B2**) (26–29, 50), a significant increase in the risk of peripheral neuropathy could only be seen in grades 3–5 [OR = 1.76, 95%CI:(1.10, 2.82), I^2^ = 0%, Z = 2.37 (*P* = 0.02); **Figure 3B2**] (26–29, 50). The corresponding funnel plots are provided in the supplement (**S Figure 1B1, B2**) (26–29, 50).

### Risk of Peripheral Sensory Neuropathy

**Figure 4:**
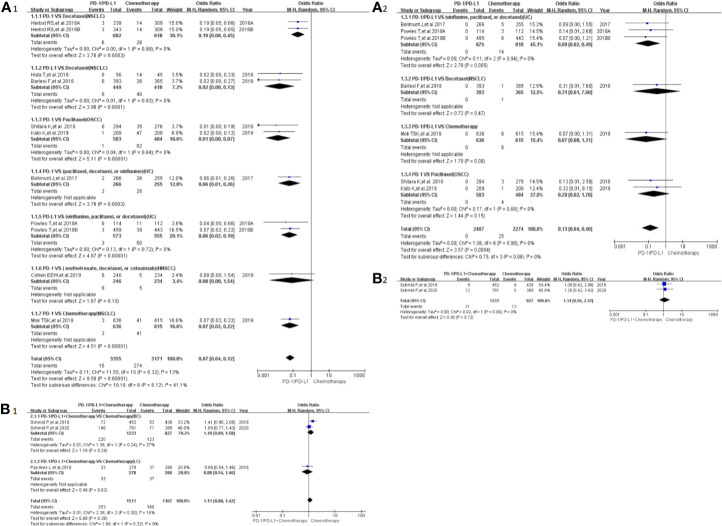
Forest plots of the risk of peripheral sensory neuropathy **(A1)** The risk of all-grade peripheral sensory neuropathy calculated by the random effect (RE) model (PD-1/PD-L1 *vs* chemotherapy): subgroup analysis was put into practice based on PD-1/PD-L1 and tumor types in both groups. **(A2)** The risk of peripheral sensory neuropathy of grades 3–5 calculated by the random effect (RE) model (PD-1/PD-L1 *vs* chemotherapy): subgroup analysis was put into practice based on PD-1/PD-L1 and tumor types in both groups. **(B1)** The risk of all-grade peripheral sensory neuropathy calculated by the random effect (RE) model (PD-1/PD-L1 + chemotherapy *vs* chemotherapy): subgroup analysis was put into practice based on tumor types in both groups. **(B2)** The risk of peripheral sensory neuropathy of grades 3–5 calculated by the random effect (RE) model (PD-1/PD-L1+ chemotherapy *vs.* chemotherapy): subgroup analysis was put into practice based on tumor types in both groups.

Peripheral sensory neuropathy was reported in 13 clinical trials (24–26, 29–34, 41, 42, 46, 50), 12 of which were included in the final meta-analysis (24–26, 29–34, 41, 46, 50). When PD-1/PD-L1 inhibitors were compared with chemotherapy, the risk of peripheral sensory neuropathy of all grades was obviously lower [OR = 0.07, 95%CI:(0.04, 0.12), I^2^ = 13%, Z = 9.50(*P <* 0.00001); **Figure 4A1**] (24, 25, 30–34, 41, 46), while similar risk trends of grades 3–5 were seen between both arms [OR = 0.13, 95%CI:(0.04, 0.40), I^2^ = 0%, Z=3.57 (*P* = 0.0004); **Figure 4A2**] (24, 30–32, 34, 46). The corresponding funnel plots are provided in the supplement (**S Figure 2A1, A2**) (24–26, 29–34, 41, 46, 50).

When PD-1/PD-L1 inhibitors plus chemotherapy were compared with chemotherapy (**Figures 4B1, B2**) (26–29, 50), no statistically significant difference was found (26, 29, 50). The corresponding funnel plots are provided in the supplement **(** **S Figure 2B1, B2**) (26, 29, 50).

### Risk of Dysgeusia

**Figure 5:**
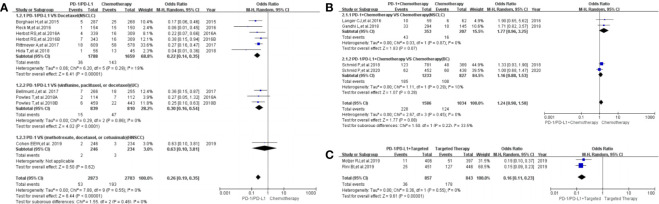
Forest plots of the risk of dysgeusia. **(A)** The risk of all-grade dysgeusia calculated by the random effect (RE) model (PD-1/PD-L1 *vs* chemotherapy): subgroup analysis was put into practice based on PD-1/PD-L1 and tumor types in both groups. **(B)** The risk of all-grade dysgeusia calculated by the random effect (RE) model (PD-1/PD-L1+ chemotherapy *vs.* chemotherapy): subgroup analysis was put into practice based on PD-1/PD-L1 and tumor types in both groups. **(C)** The risk of all-grade dysgeusia calculated by the random effect (RE) model (PD-1/PD-L1+ targeted *vs.* targeted therapy): subgroup analysis was put into practice based on tumor types in both groups.

Dysgeusia was reported in 16 clinical trials (22, 23, 25, 26, 32–37, 41–43, 45, 47, 50), 14 of which were included in the final meta-analysis (22, 23, 25, 26, 32–37, 41, 43, 47, 50). When PD-1/PD-L1 inhibitors were compared with chemotherapy, the risk of dysgeusia of all grades was obviously lower [OR=0.26, 95%CI:(0.19, 0.35), I^2^ = 0%, Z = 8.44 (*P <* 0.00001); **Figure 5A**] (25, 32–35, 37, 41, 43), especially for subgroups relating to NSCLC and UC (32–35, 37, 41, 43). The corresponding funnel plot is provided in the supplement (**S Figure 3A1**) (25, 32–35, 37, 41, 43).

When PD-1/PD-L1 inhibitors plus chemotherapy were compared with chemotherapy (**Figure 5B**), no statistically significant difference was noted [OR = 1.24, 95%CI:(0.98, 1.58), I^2^ = 0%, Z = 1.77 (*P* = 0.08); **Figure 5B**] (26, 36, 47, 50). The corresponding funnel plot is provided in the supplement (**S Figure 3A2**) (26, 36, 47, 50).

When PD-1/PD-L1 inhibitors plus targeted therapy were compared with targeted therapy (**Figure 5C**), the risk of dysgeusia of all grades was obviously lower [OR = 0.16, 95%CI:(0.11, 0.23), I^2^ = 0%, Z = 9.61 (*P <* 0.00001); **Figure 5C**] (22, 23). The corresponding funnel plot is provided in the supplement (**S Figure 3A3**) (22, 23).

The risk of dysgeusia grades 3–5 could not be analyzed in the meta-analysis due to the limited data available in the included trials (23, 47).

### Risk of Paraesthesia

**Figure 6:**
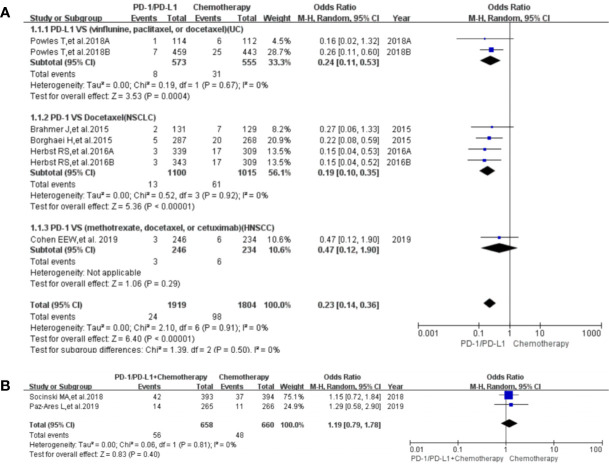
Forest plots of the risk of paraesthesia. **(A)** The risk of all-grade paraesthesia calculated by the random effect (RE) model (PD-1/PD-L1 *vs.* chemotherapy): subgroup analysis was put into practice based on PD-1/PD-L1 and tumor types in both groups. **(B)** The risk of all-grade dysgeusia calculated by the random effect (RE) model (PD-1/PD-L1 + chemotherapy *vs.* chemotherapy).

Paraesthesia was reported in eight clinical trials (25, 28, 32, 41–44, 49), seven of which were included in the final meta-analysis (25, 28, 32, 41, 43, 44, 49). When PD-1/PD-L1 inhibitors were compared with chemotherapy, the risk of paraesthesia of all grades was obviously lower [OR = 0.23, 95%CI:(0.14, 0.36), I^2^ = 0%, Z = 6.40 (*P <* 0.00001); **Figure 6A**] (25, 28, 32, 41, 43, 44, 49), especially for subgroups relating to NSCLC and UC (32, 41, 43, 44). No heterogeneity was found (**Figure 6A**, I^2^ = 0%) (25, 28, 32, 41, 43, 44, 49). The corresponding funnel plot is provided in the supplement (**S Figure 3B1**) (25, 28, 32, 41, 43, 44, 49).

When PD-1/PD-L1 inhibitors plus chemotherapy were compared with chemotherapy, no statistically significant difference was found for paraesthesia of all grades [OR = 1.19, 95%CI:(0.79, 1.78), I^2^ = 0%, Z = 0.83 (*P* = 0.40); **Figure 6B** **) (** 28, 49). The corresponding funnel plot is provided in the supplement (**S Figure 3B2** **)** (28, 49).

### Risk of Headache

Headache was reported in 17 articles, involving 12 clinical trials (22, 23, 25, 26, 34, 41, 43, 47, 48, 51–57). When PD-1/PD-L1 inhibitors were compared with chemotherapy, no statistically significant differences were found in terms of all grade and grades 3–5 headache (**S Figure 4A1, A2**) (25, 34, 41, 43). A similar risk trend was also noted when PD-1/PD-L1 inhibitors plus others were compared with the control groups (**S Figure 4B, C2, D1, D2**) (22, 26, 47, 48, 51, 54).

When PD-1/PD-L1 inhibitors plus targeted therapy were compared with targeted therapy, the risk of headache of all grades was obviously higher [OR = 1.43, 95%CI:(1.09, 1.86), I^2^ = 0%, Z=2.62 (*P* = 0.0009); **Supplementary Figure 4C1**) (22, 23, 48). The corresponding funnel plots are provided in the supplement (**S Figure 5**) (22, 23, 25, 26, 34, 41, 43, 47, 48, 51, 54).

### Risk of Dizziness

Dizziness was reported in 12 articles, involving 11 clinical trials (22, 25, 34, 36, 38, 41–44, 47, 51, 52). According to different treatment regimens, we divided all included clinical trials into four groups to investigate the risk of dizziness of all grades and grades 3–5. However, no statistically significant differences were noted (**Supplementary Figure 6**) (25, 34, 36, 38, 41–44, 47, 51). The corresponding funnel plots are provided in the supplement (**S Figure 7**) (25, 34, 36, 38, 41–44, 47, 51).

### Risk of Rarely Reported Neurologic Toxicities

Other types of neurological toxicities were reported in a limited number of studies, including peripheral motor neuropathy (51), Guillain–Barré syndrome (**Supplementary Figure 8A,B**) (25, 27, 33, 42, 51), polyneuropathy (**Supplementary Figure 8C**) (10, 25, 51), neurotoxicity (25). For Guillain–Barré syndrome and polyneuropathy, compared with chemotherapy, a statistically significant reduction in their associated risk was only observed in polyneuropathy [OR = 0.12, 95%CI:(0.01, 0.940, I^2^ = 0%, Z = 2.02 (P = 0.04); **Supplementary Figure 8C**) (10, 25, 51). The corresponding funnel plots are provided in the supplement (**Supplementary Figure 9** **) (** 10, 25, 27, 33, 42, 51). Due to the unavailability of relevant data regarding the other two neurological toxicities (neurotoxicity and peripheral motor neuropathy), they could not be included in the meta-analysis (25, 51).

## Discussion

Most of the neurological toxicities caused by PD-1/PD-L1 inhibitors might be presented as low-grade appearances, with the potential to involve any aspect of the central or peripheral nervous system (7, 8). As more and more clinical trials reporting the efficacy and safety of PD-1/PD-L1 in cancer patients are being conducted, the reporting of drug-induced neurological toxicities has gradually increased (1, 2, 22–57). In order to clarify the relationship between PD-1/PD-L1 inhibitors and the risk of neurological toxicities in cancer patients, this meta-analysis was designed. It was the first time that neurological toxicities were comprehensively investigated through a meta-analytic approach instead of case reports and reviews (1, 5–14). It would be helpful in guiding anti-PD-1 and anti-PD-L1 immunotherapy.

Thirty-six articles, including 31 clinical trials with available data regarding neurological toxicities, were included in our study (22–57). Among the included clinical trials, lung cancer-related clinical trials accounted for the largest proportion (N = 17) (24, 27–30, 33, 35–37, 39–41, 43, 44, 47, 49, 55–57). Of note, the majority of the included clinical trials were of high quality (low risk of bias) (22–57). Therefore, the conclusion drawn from those data would be of higher credibility.

In our meta-analysis, we noted that the risk of all-grade neurological toxicities in the PD-1/PD-L1 inhibitors group was lower compared to the chemotherapy arm. These neurological toxicities included peripheral neuropathy, peripheral sensory neuropathy, dysgeusia, paraesthesia, and polyneuropathy (**Figure 3A1**, **4A1**, **5A1**, **6A1**, **S Figure 4A1**, **8C**). A similar observation was noted regarding peripheral neuropathy and peripheral sensory neuropathy of grades 3–5 (**Figure 3A2**, **4A2**) (10, 22–47, 49–51). These findings highlight the need to pay more attention to the risk of neurological toxicities associated with chemotherapy in clinical practice, especially for docetaxel (26, 30–32, 34, 40, 41, 43, 44, 46). The subgroup analyses suggested that the encountered high heterogeneity in our analyses (I^2^=62%) might be related to the NSCLC subgroup (I^2^ = 75%, **Figure 3A1**) (26, 30, 40). In addition, the treatment plans involved in the three NSCLC clinical trials included in the comprehensive analysis belonged to different treatment lines (first, second, or third line); this probably might be a potential contributor to the heterogeneity of the result (I^2^ = 75%, **Figure 3A1**) (26, 30, 40). That being said, no obvious risk of publication bias was found from the corresponding funnel plots (**Supplementary Figure 1A1**, **2A1**, **3A1, B1**, **5A1**, **9C** **)**. Interestingly, for headache, dizziness, and Guillain-Barré syndrome, the risk was found to be of no significance (**Supplementary Figure 4A**, **6A**, **8A**) (22, 23, 25–27, 33, 34, 36, 38, 41–44, 47, 48, 51–57), which meant that the risk trend of the aforementioned three neurological toxicities caused by PD-1/PD-L1 inhibitors was similar to that of the chemotherapy group. This finding is novel and has not been reported nor investigated by other studies in the literature.

Furthermore, Guillain–Barré syndrome was reported in five PD-1/PD-L1 groups (all cases were reported in the PD-1/PD-L1 group), while the incidence rate of the control groups was 0 (25, 27, 33, 42, 51). No statistically significant difference was noted and this could be attributed to the small number of included trials and the sensitivity of the analysis method (25, 27, 33, 42, 51). That being said, we cannot rule out the possibility that Guillain–Barré syndrome is a unique neurological toxicity of PD-1/PD-L1 inhibitors. Despite the fact that our analyses revealed some statistically insignificant results; however, the reported risks should not be ignored in clinical practice, and more attention should be paid to those fatal and rare reported neurological toxicities (25, 27, 33, 42, 51). These results might be of significant value in clinical practice. Once Guillain-Barré syndrome happened, we should first consider its associations with PD-1/PD-L1 inhibitors (25, 27, 33, 42, 51).

When PD-1/PD-L1 inhibitors plus chemotherapy were compared with chemotherapy, the trends in the risk of all-grade neurological toxicities increased without statistically significant differences (**Figure 3B1**, **4B1**, **5B**, **6B**, **Supplementary Figure 4B**, **6B**) (26–29, 36, 47, 49, 50). Statistically significant results were only found in terms of peripheral neuropathy of grades 3–5, especially for the breast cancer subgroup [OR = 1.76, 95%CI:(1.10, 2.82), I^2^ = 0%, Z = 2.37 (P = 0.02); **Figure 3B2**] (26–29, 50). In order to draw a definite conclusion, more relevant clinical trials are still warranted to be conducted, and sufficient subgroup analyses still need to be carried out.

When PD-1/PD-L1 inhibitors plus targeted therapy were compared with targeted therapy (**Figure 5C**), the risk of all-grade dysgeusia was notably lower than that of the control group [OR = 0.16, 95%CI:(0.11, 0.23), I^2^ = 0%, Z = 9.61 (*P <* 0.00001); **Figure 5C**) (22, 23). On the contrary, the risk of all-grade headache was increased compared to the targeted therapy group [OR = 1.43, 95%CI:(1.09, 1.86), I^2^ = 0%, Z = 2.62 (*P* = 0.0009); **Supplementary Figure 4C1**] (22, 23, 48). However, the number of analyzed studies was low, and thus, a definite conclusion could not be reached (22, 23, 48). This was also observed when PD-1/PD-L1 inhibitors plus CTLA-4 were compared with CTLA-4 analog **Supplementary Figure 4D1, D2**, **6C**, **8B**). Eventually, based on the low number of analyzed studies and the minimal data reported in these studies, our findings should be interpreted with caution, and no clinical recommendations should be implemented from these data.

## Strengths and Limitations

### Strengths

This article was designed according to the PRISMA guidelines. The literature searching process was carried out in accordance with the PICOS principle. We strictly limited the selection criteria to clinical trials and checked the accuracy of the extracted data carefully. The quality of the majority of the included trials was high. Subgroup analyses were put into practice as much as possible. Therefore, our meta-analysis provided a much more reliable evaluation of the relationship between PD-1/PD-L1 inhibitors and the associated risk of neurological toxicities in cancer patients compared to available evidence in the literature.

### Limitations

First, compared with the control group, all the analysis results just showed the relative risk of neurological toxicities in cancer patients. Even when the associated risk of neurological toxicity was lower than that of the control group, it did not mean that PD-1/PD-L1 would not cause neurological toxicity in the experimental group. Second, the low number of studies that reported the data of certain neurological toxicities, along with the unavailability of relevant data, made it difficult to conduct a meta-analysis in this regard. Therefore, a definite conclusion could not be reached.

## Conclusion

Our comprehensive review showed that PD-1/PD-L1 inhibitors alone exhibited lower neurological toxicities than chemotherapy. However, in terms of headache, dizziness, and Guillain–Barré syndrome, the risk trends were similar between both interventions. Regarding PD-1/PD-L1 inhibitors plus chemotherapy, the risk of neurological toxicities would be increased, especially for peripheral neuropathy of grades 3–5.

## Data Availability Statement

The original contributions presented in the study are included in the article/**Supplementary Material**. Further inquiries can be directed to the corresponding authors.

## Author Contributions

The corresponding authors (YS and GS) had the right to deal with all the data and were responsible for the decision to submit this manuscript for publication. YT, AG, SW, SZ, and XY had the full data of the manuscript. YT, AG, SW, and SZ were responsible for checking and evaluating the quality of the data and included studies. YT was assigned to write the text of this manuscript. All authors contributed to the article and approved the submitted version.

## Funding

This study was funded by the Academic Promotion Program of Shandong First Medical University (2019QL025; YS), Natural Science Foundation of Shandong Province (ZR2019MH042; YS), Jinan Science and Technology Program (201805064; YS), and the National Science and Technology Major Project of the Ministry of Science and Technology of China (2020ZX09201025; GS).

## Conflict of Interest

The authors declare that the research was conducted in the absence of any commercial or financial relationships that could be construed as a potential conflict of interest.

## Supplementary Material

Supplementary Figure 1 Funnel plots of the risk of peripheral neuropathy. **(A1)** The risk of all-grade peripheral neuropathy calculated by the fixed effect (FE) model (PD-1/PD-L1 *vs.* chemotherapy): subgroup analysis was put into practice based on PD-1/PD-L1 and tumor types in both groups. **(A2)** The risk of peripheral neuropathy of grades 3–5 calculated by the fixed effect (FE) model (PD-1/PD-L1 *vs*. chemotherapy): subgroup analysis was put into practice based on PD-1/PD-L1 and tumor types in both groups. **(B1)** The risk of all-grade peripheral neuropathy calculated by the fixed effect (FE) model (PD-1/PD-L1 + chemotherapy *vs.* chemotherapy): subgroup analysis was put into practice based on tumor types in both groups. **(B2)** The risk of peripheral neuropathy of grades 3–5 calculated by the fixed effect (FE) model (PD-1/PD-L1 + chemotherapy *vs.* chemotherapy): subgroup analysis was put into practice based on tumor types in both groups.

Supplementary Figure 2 Funnel plots of the risk of peripheral sensory neuropathy. **(A1)** The risk of all-grade peripheral sensory neuropathy calculated by the fixed effect (FE) model (PD-1/PD-L1 *vs.* chemotherapy): subgroup analysis was put into practice based on PD-1/PD-L1 and tumor types in both groups. **(A2)** The risk of peripheral sensory neuropathy of grades 3–5 calculated by the fixed effect (FE) model (PD-1/PD-L1 *vs*. chemotherapy): subgroup analysis was put into practice based on PD-1/PD-L1 and tumor types in both groups. **(B1)** The risk of all-grade peripheral sensory neuropathy calculated by the fixed effect (FE) model (PD-1/PD-L1 + chemotherapy *vs.* chemotherapy): subgroup analysis was put into practice based on tumor types in both groups. **(B2)** The risk of peripheral sensory neuropathy of grades 3–5 calculated by the fixed effect (FE) model (PD-1/PD-L1 + chemotherapy *vs.* chemotherapy): subgroup analysis was put into practice based on tumor types in both groups.

Supplementary Figure 3
**(A)** Funnel plots of the risk of dysgeusia. **(A1)** The risk of all-grade dysgeusia calculated by the fixed effect (FE) model (PD-1/PD-L1 *vs.* chemotherapy): subgroup analysis was put into practice based on PD-1/PD-L1 and tumor types in both groups. **(A2)** The risk of all-grade dysgeusia calculated by the fixed effect (FE) model. (PD-1/PD-L1 + chemotherapy *vs.* chemotherapy): subgroup analysis was put into practice based on PD-1/PD-L1 and tumor types in both groups. **(A3)** The risk of all-grade dysgeusia calculated by the fixed effect (FE) model. (PD-1/PD-L1 + targeted *vs*. targeted therapy): subgroup analysis was put into practice based on tumor types in both groups. **(B)** Funnel plots of the risk of paraesthesia. **(B1)** The risk of all-grade paraesthesia calculated by the fixed effect (FE) model (PD-1/PD-L1 *vs.* chemotherapy): subgroup analysis was put into practice based on PD-1/PD-L1 and tumor types in both groups. **(B2)** The risk of all-grade paraesthesia calculated by the fixed effect (FE) model (PD-1/PD-L1 + chemotherapy *vs.* chemotherapy).

Supplementary Figure 4 Forest plots of the risk of headache. **(A1)** The risk of all-grade headache calculated by the random effect (RE) model (PD-1/PD-L1 *vs.* chemotherapy): subgroup analysis was put into practice based on tumor types in both groups. **(A2)** The risk of headache of grades 3–5 calculated by the random effect (RE) model (PD-1/PD-L1 *vs.* chemotherapy). **(B)** The risk of all-grade headache calculated by the random effect (RE) model (PD-1/PD-L1 + targeted *vs.* targeted chemotherapy): subgroup analysis was put into practice based on PD-1 or PD-L1. **(C1)** The risk of all-grade headache calculated by the random effect (RE) model (PD-1/PD-L1 + targeted *vs.* targeted therapy): subgroup analysis was put into practice based on PD-1/PD-L1 and tumor types in both groups. **(C2)** The risk of headache of grades 3–5 calculated by the random effect (RE) model (PD-1/PD-L1 + targeted *vs.* targeted therapy). **(D1)** The risk of all-grade headache calculated by the random effect (RE) model (PD-1/PD-L1 + CTLA-4 *vs.* CTLA-4). **(D2)** The risk of headache of grades 3–5 calculated by the random effect (RE) model (PD-1/PD-L1 + CTLA-4 *vs*. CTLA-4).

Supplementary Figure 5 Funnel plots of the risk of headache. **(A1)** The risk of all-grade headache calculated by the fixed effect **(FE)** model (PD-1/PD-L1 *vs*. chemotherapy): subgroup analysis was put into practice based on tumor types in both groups. **(A2)** The incidence risk of headache of grades 3–5 calculated by the fixed effect **(FE)** model (PD-1/PD-L1 *vs.* chemotherapy). **(B)** The risk of all-grade headache calculated by the fixed effect **(FE)** model (PD-1/PD-L1 + targeted *vs.* targeted therapy): subgroup analysis was put into practice based on PD-1 or PD-L1. **(C1)** The risk of all-grade headache calculated by the fixed effect **(FE)** model (PD-1/PD-L1 + targeted *vs.* targeted therapy): subgroup analysis was put into practice based on PD-1/PD-L1 and tumor types in both groups. **(C2)** The risk of headache of grades 3–5 calculated by the fixed effect **(FE)** model (PD-1/PD-L1 + targeted *vs.* targeted therapy). (**D1**) The risk of all-grade headache calculated by the fixed effect **(FE)** model (PD-1/PD-L1 + CTLA-4 *vs.* CTLA-4). (**D2**) The risk of headache of grades 3–5 calculated by the fixed effect **(FE)** model (PD-1/PD-L1 + CTLA-4 *vs.* CTLA-4).

Supplementary Figure 6 Forest plots of the risk of dizziness. **(A1)** The risk of all-grade dizziness calculated by the random effect (RE) model (PD-1/PD-L1 *vs.* chemotherapy): subgroup analysis was put into practice based on PD-1/PD-L1 and tumor types in both groups. **(A2)** The risk of dizziness of grades 3–5 calculated by random effect (RE) model (PD-1/PD-L1 *vs.* chemotherapy). **(B)** The risk of all-grade dizziness calculated by the random effect (RE) model (PD-1/PD-L1 +c hemotherapy *vs*. chemotherapy). **(C)** The risk of all-grade dizziness calculated by the random effect (RE) model (PD-1/PD-L1 + CTLA-4 *vs*. CTLA-4).

Supplementary Figure 7 Funnel plots of the risk of dizziness. **(A1)** The risk of all-grade dizziness calculated by the fixed effect **(FE)** model (PD-1/PD-L1 *vs*. chemotherapy): subgroup analysis was put into practice based on PD-1/PD-L1 and tumor types in both groups. (A2) The risk of dizziness of grades 3–5 calculated by the fixed effect **(FE)** model (PD-1/PD-L1 *vs.* chemotherapy). **(B)** The risk of all-grade dizziness calculated by the fixed effect **(FE)** model (PD-1/PD-L1 + chemotherapy *vs.* chemotherapy). **(C)** The risk of all-grade dizziness calculated by the fixed effect **(FE)** model (PD-1/PD-L1 + CTLA-4 *vs*. CTLA-4).

Supplementary Figure 8 Forest plots of the risk of rarely reported neurological toxicities. **(A1)** The risk of all-grade Guillain–Barré Syndrome calculated by the random effect (RE) model (PD-1/PD-L1 *vs*. chemotherapy): subgroup analysis was put into practice based on PD-1/PD-L1 and tumor types in both groups. **(A2)** The risk of Guillain–Barré Syndrome of grades 3–5 calculated by the random effect (RE) model (PD-1/PD-L1 *vs.* chemotherapy). **(B)** The risk of all-grade Guillain–Barré Syndrome calculated by the random effect (RE) model (PD-1/PD-L1 + CTLA-4 *vs*. CTLA-4). **(C)** The risk of all-grade polyneuropathy calculated by the random effect (RE) model (PD-1/PD-L1 + CTLA-4 *vs.* CTLA-4).

Supplementary Figure 9 Funnel plots of the risk of rarely reported neurological toxicities. **(A1)** The risk of all-grade Guillain–Barré Syndrome calculated by the fixed effect **(FE)** model (PD-1/PD-L1 *vs*. chemotherapy): subgroup analysis was put into practice based on PD-1/PD-L1 and tumor types in both groups. **(A2)** The risk of Guillain–Barré Syndrome of grades 3–5 calculated by the fixed effect **(FE)** model (PD-1/PD-L1 *vs.* chemotherapy). **(B)** The risk of all-grade Guillain–Barré Syndrome calculated by the fixed effect **(FE)** model (PD-1/PD-L1 + CTLA-4 *vs.* CTLA-4). **(C)** The risk of all-grade polyneuropathy calculated by the fixed effect **(FE)** model (PD-1/PD-L1 + CTLA-4 *vs*. CTLA-4).

## Abbreviations

PRISMA, Preferred Reporting Items for Systematic Reviews and Meta-Analyses; PICOS, Participants, Interventions, Comparisons, Outcomes, and Study design; PD-1, Programmed Cell Death-1; PD-L1, Programmed Cell Death Ligand 1; HR, Hazard Ratios; OR. Odds Ratio; RD, Risk Difference; CI, Confidence Interval; RE, Random Effect; NSCLC, Non-Small Cell Lung Cancer; SCLC, Small Cell Lung Cancer; OSCC, Esophageal Squamous Cell Carcinoma; HNSCC, Head and Neck Squamous Cell Carcinoma; UC, Urothelial Cancer; BC, Breast Cancer; RCC, Renal Cell Carcinoma; NOS, Newcastle-Ottawa scale.
