# Supplementary figures and images for: Immune-Related Neurological Toxicities of PD-1/PD-L1 Inhibitors in Cancer Patients: A Systematic Review and Meta-Analysis
Source: Front Immunol. 2020 Dec 18;11:595655. doi: 10.3389/fimmu.2020.595655 (PMC7775530; doi:10.3389/fimmu.2020.595655)

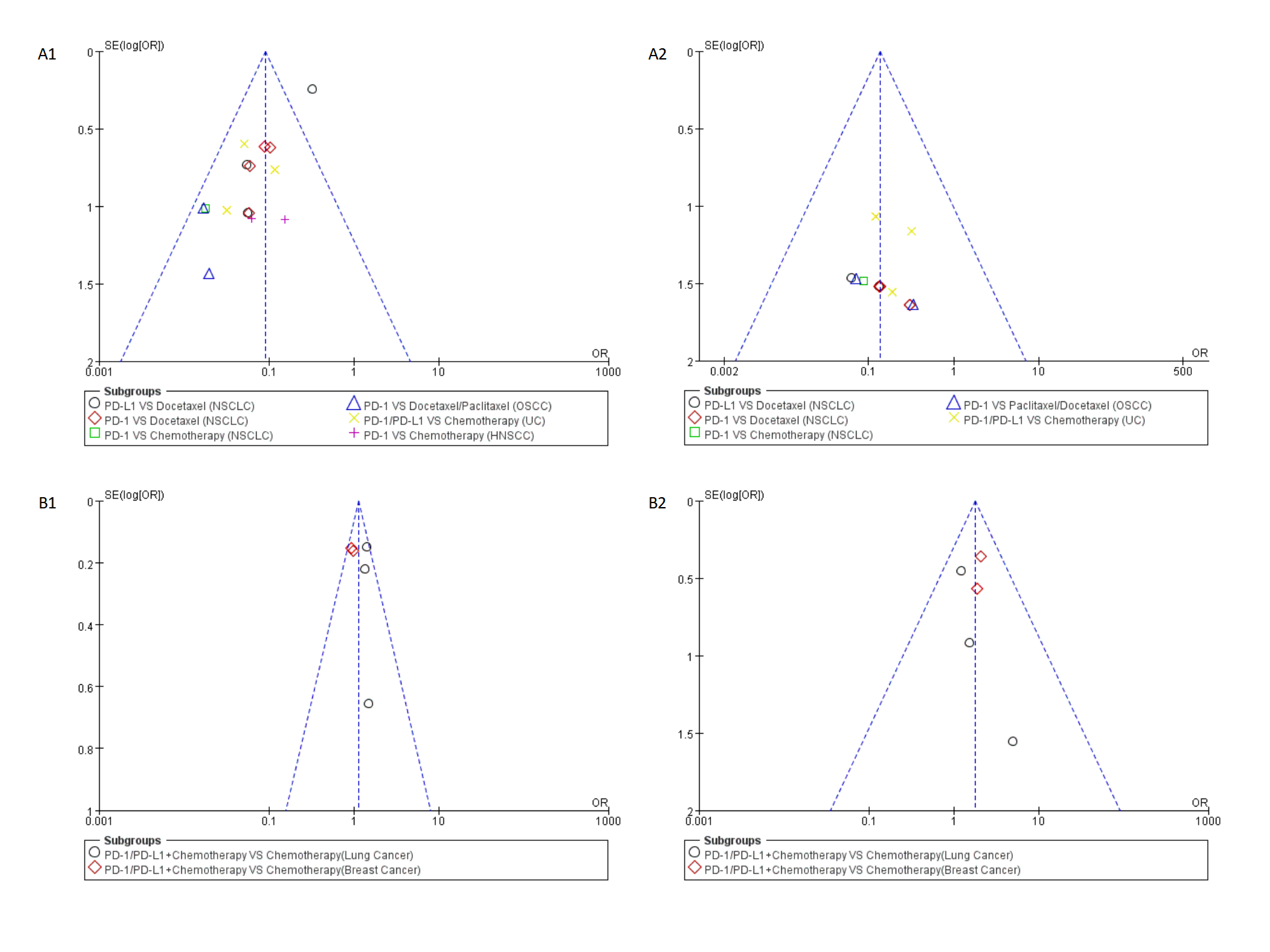

Supplement: Supplementary Figure 1 — Funnel plots of the risk of peripheral neuropathy. (A1) The risk of all-grade peripheral neuropathy calculated by the fixed effect (FE) model (PD-1/PD-L1 vs. chemotherapy): subgroup analysis was put into practice based on PD-1/PD-L1 and tumor types in both groups. (A2) The risk of peripheral neuropathy of grades 3–5 calculated by the fixed effect (FE) model (PD-1/PD-L1 vs. chemotherapy): subgroup analysis was put into practice based on PD-1/PD-L1 and tumor types in both groups. (B1) The risk of all-grade peripheral neuropathy calculated by the fixed effect (FE) model (PD-1/PD-L1 + chemotherapy vs. chemotherapy): subgroup analysis was put into practice based on tumor types in both groups. (B2) The risk of peripheral neuropathy of grades 3–5 calculated by the fixed effect (FE) model (PD-1/PD-L1 + chemotherapy vs. chemotherapy): subgroup analysis was put into practice based on tumor types in both groups. [file Image_1.tif]

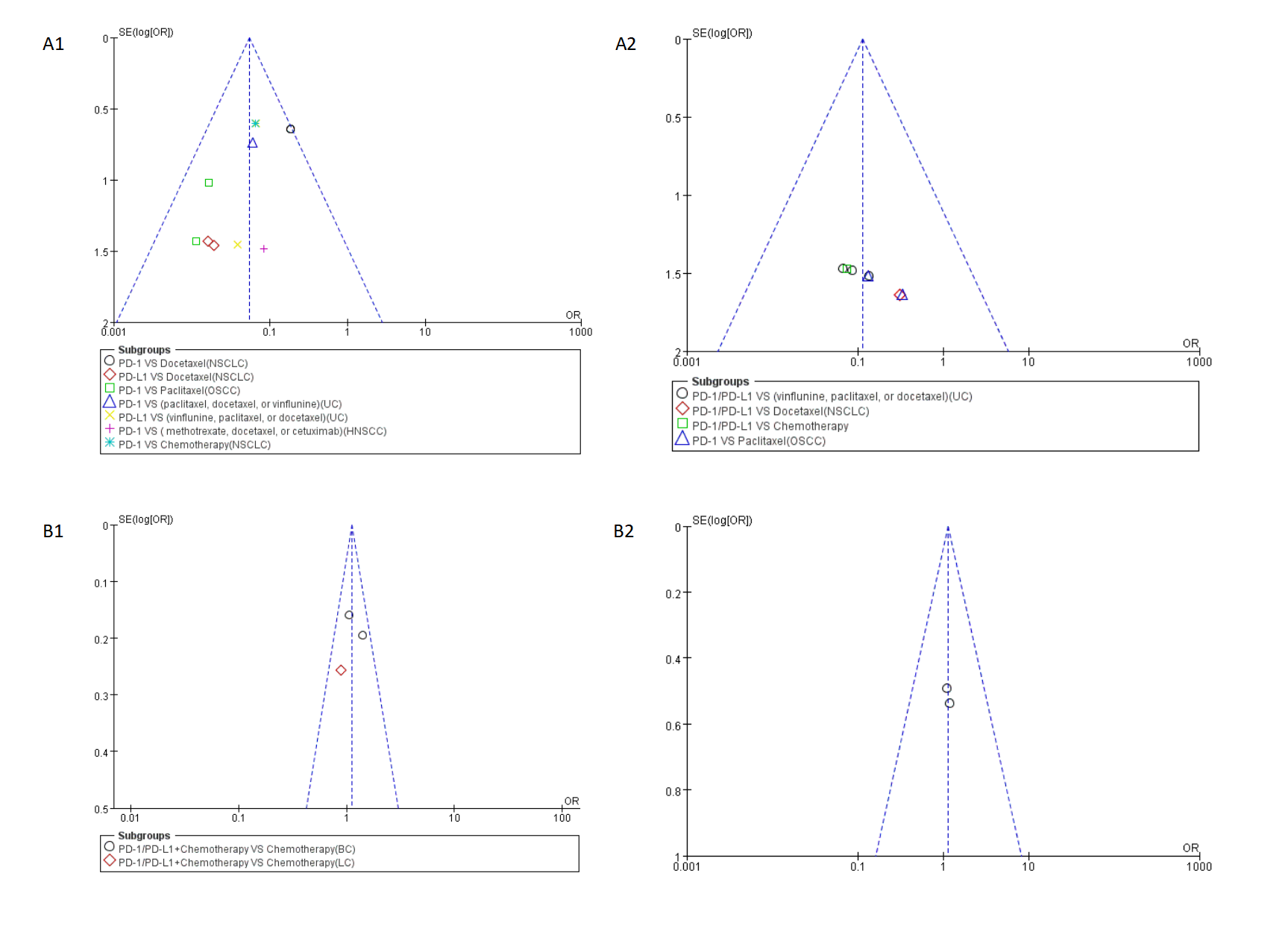

Supplement: Supplementary Figure 2 — Funnel plots of the risk of peripheral sensory neuropathy. (A1) The risk of all-grade peripheral sensory neuropathy calculated by the fixed effect (FE) model (PD-1/PD-L1 vs. chemotherapy): subgroup analysis was put into practice based on PD-1/PD-L1 and tumor types in both groups. (A2) The risk of peripheral sensory neuropathy of grades 3–5 calculated by the fixed effect (FE) model (PD-1/PD-L1 vs. chemotherapy): subgroup analysis was put into practice based on PD-1/PD-L1 and tumor types in both groups. (B1) The risk of all-grade peripheral sensory neuropathy calculated by the fixed effect (FE) model (PD-1/PD-L1 + chemotherapy vs. chemotherapy): subgroup analysis was put into practice based on tumor types in both groups. (B2) The risk of peripheral sensory neuropathy of grades 3–5 calculated by the fixed effect (FE) model (PD-1/PD-L1 + chemotherapy vs. chemotherapy): subgroup analysis was put into practice based on tumor types in both groups. [file Image_2.tif]

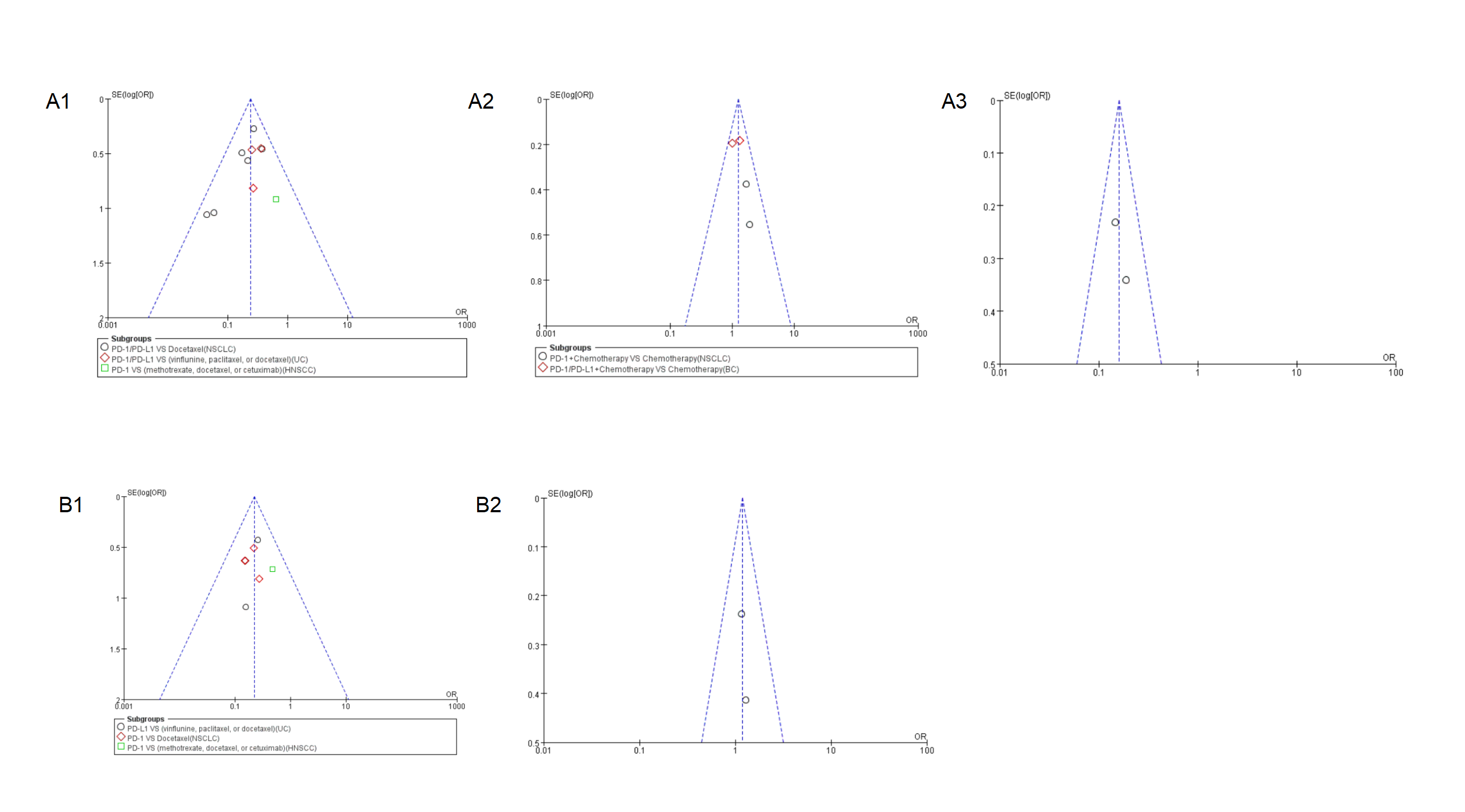

Supplement: Supplementary Figure 3 — (A) Funnel plots of the risk of dysgeusia. (A1) The risk of all-grade dysgeusia calculated by the fixed effect (FE) model (PD-1/PD-L1 vs. chemotherapy): subgroup analysis was put into practice based on PD-1/PD-L1 and tumor types in both groups. (A2) The risk of all-grade dysgeusia calculated by the fixed effect (FE) model. (PD-1/PD-L1 + chemotherapy vs. chemotherapy): subgroup analysis was put into practice based on PD-1/PD-L1 and tumor types in both groups. (A3) The risk of all-grade dysgeusia calculated by the fixed effect (FE) model. (PD-1/PD-L1 + targeted vs. targeted therapy): subgroup analysis was put into practice based on tumor types in both groups. (B) Funnel plots of the risk of paraesthesia. (B1) The risk of all-grade paraesthesia calculated by the fixed effect (FE) model (PD-1/PD-L1 vs. chemotherapy): subgroup analysis was put into practice based on PD-1/PD-L1 and tumor types in both groups. (B2) The risk of all-grade paraesthesia calculated by the fixed effect (FE) model (PD-1/PD-L1 + chemotherapy vs. chemotherapy). [file Image_3.tif]

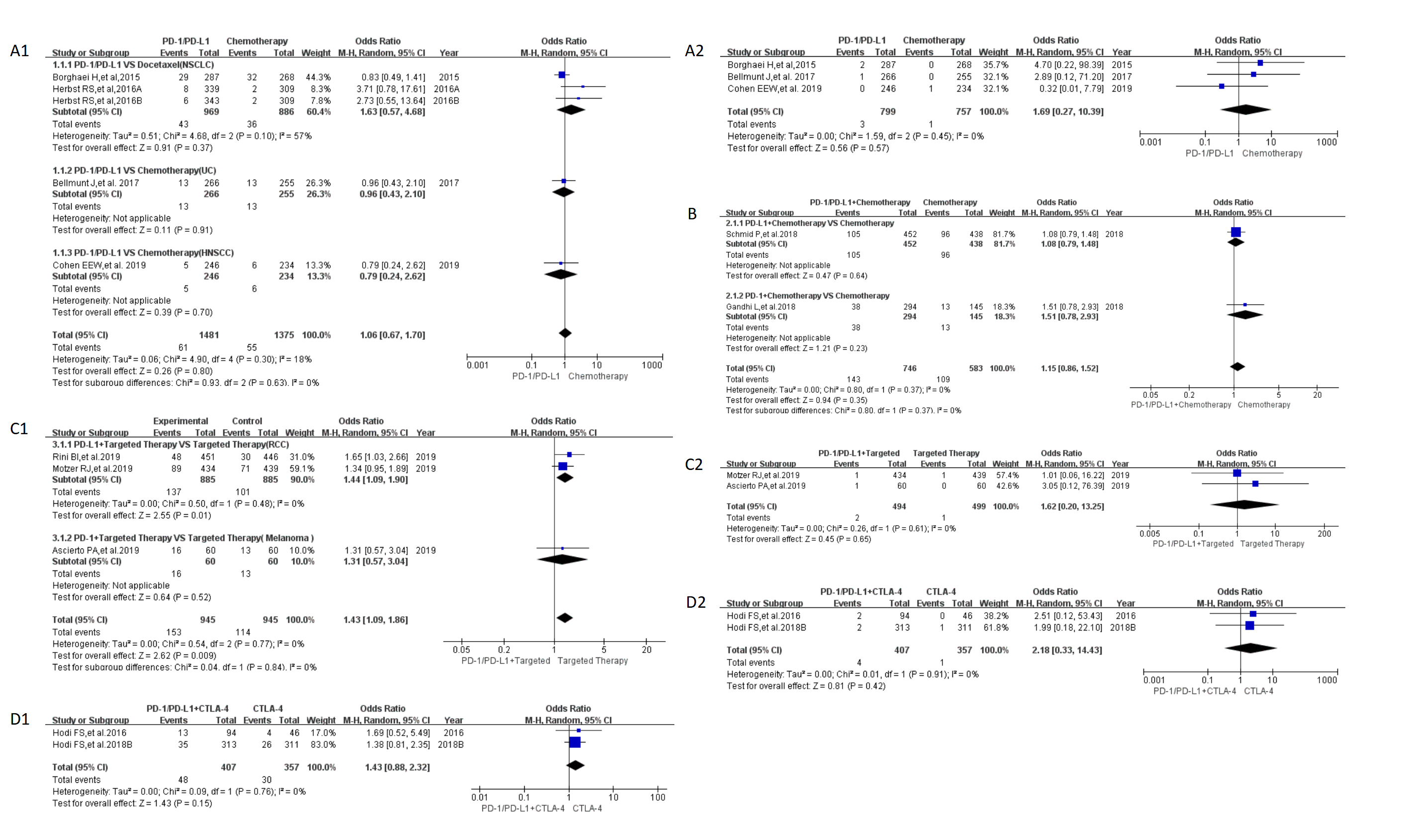

Supplement: Supplementary Figure 4 — Forest plots of the risk of headache. (A1) The risk of all-grade headache calculated by the random effect (RE) model (PD-1/PD-L1 vs. chemotherapy): subgroup analysis was put into practice based on tumor types in both groups. (A2) The risk of headache of grades 3–5 calculated by the random effect (RE) model (PD-1/PD-L1 vs. chemotherapy). (B) The risk of all-grade headache calculated by the random effect (RE) model (PD-1/PD-L1 + targeted vs. targeted chemotherapy): subgroup analysis was put into practice based on PD-1 or PD-L1. (C1) The risk of all-grade headache calculated by the random effect (RE) model (PD-1/PD-L1 + targeted vs. targeted therapy): subgroup analysis was put into practice based on PD-1/PD-L1 and tumor types in both groups. (C2) The risk of headache of grades 3–5 calculated by the random effect (RE) model (PD-1/PD-L1 + targeted vs. targeted therapy). (D1) The risk of all-grade headache calculated by the random effect (RE) model (PD-1/PD-L1 + CTLA-4 vs. CTLA-4). (D2) The risk of headache of grades 3–5 calculated by the random effect (RE) model (PD-1/PD-L1 + CTLA-4 vs. CTLA-4). [file Image_4.tif]

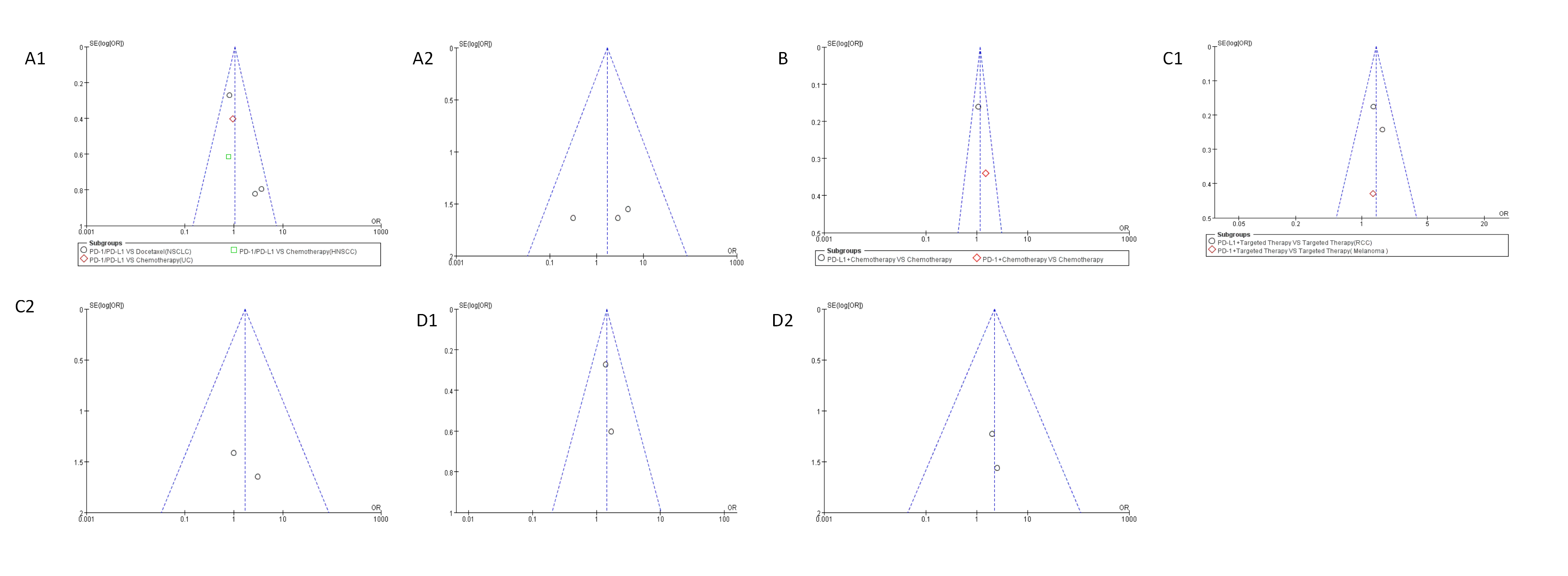

Supplement: Supplementary Figure 5 — Funnel plots of the risk of headache. (A1) The risk of all-grade headache calculated by the fixed effect (FE) model (PD-1/PD-L1 vs. chemotherapy): subgroup analysis was put into practice based on tumor types in both groups. (A2) The incidence risk of headache of grades 3–5 calculated by the fixed effect (FE) model (PD-1/PD-L1 vs. chemotherapy). (B) The risk of all-grade headache calculated by the fixed effect (FE) model (PD-1/PD-L1 + targeted vs. targeted therapy): subgroup analysis was put into practice based on PD-1 or PD-L1. (C1) The risk of all-grade headache calculated by the fixed effect (FE) model (PD-1/PD-L1 + targeted vs. targeted therapy): subgroup analysis was put into practice based on PD-1/PD-L1 and tumor types in both groups. (C2) The risk of headache of grades 3–5 calculated by the fixed effect (FE) model (PD-1/PD-L1 + targeted vs. targeted therapy). (D1) The risk of all-grade headache calculated by the fixed effect (FE) model (PD-1/PD-L1 + CTLA-4 vs. CTLA-4). (D2) The risk of headache of grades 3–5 calculated by the fixed effect (FE) model (PD-1/PD-L1 + CTLA-4 vs. CTLA-4). [file Image_5.tif]

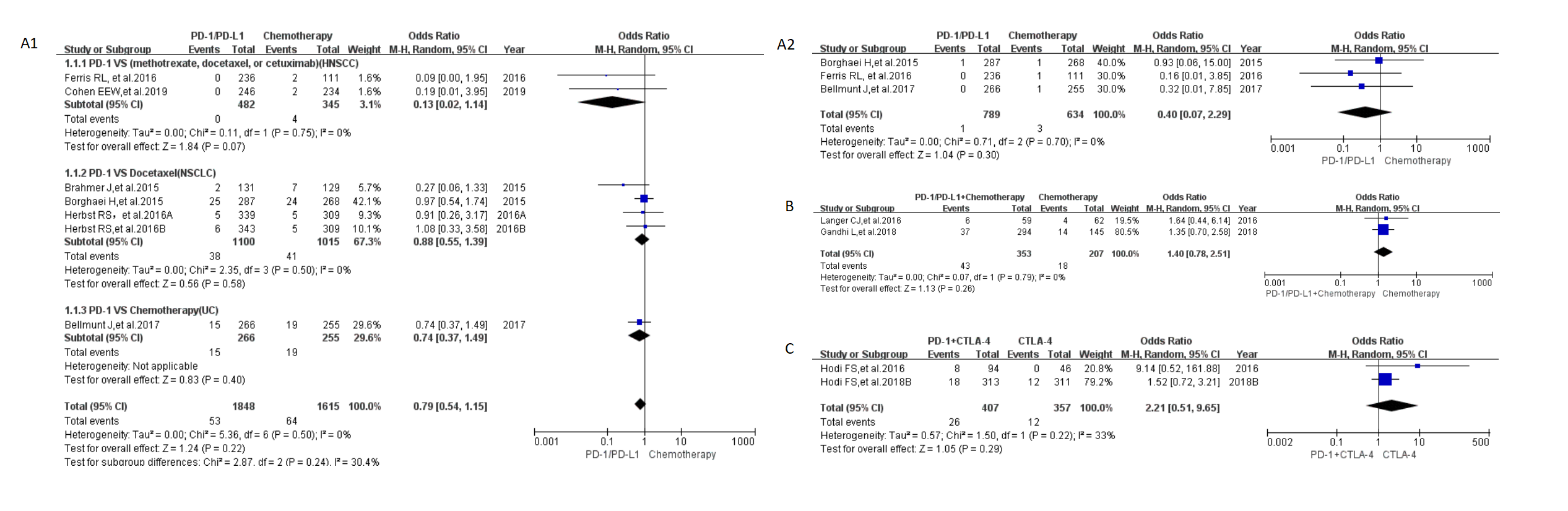

Supplement: Supplementary Figure 6 — Forest plots of the risk of dizziness. (A1) The risk of all-grade dizziness calculated by the random effect (RE) model (PD-1/PD-L1 vs. chemotherapy): subgroup analysis was put into practice based on PD-1/PD-L1 and tumor types in both groups. (A2) The risk of dizziness of grades 3–5 calculated by random effect (RE) model (PD-1/PD-L1 vs. chemotherapy). (B) The risk of all-grade dizziness calculated by the random effect (RE) model (PD-1/PD-L1 +c hemotherapy vs. chemotherapy). (C) The risk of all-grade dizziness calculated by the random effect (RE) model (PD-1/PD-L1 + CTLA-4 vs. CTLA-4). [file Image_6.tif]

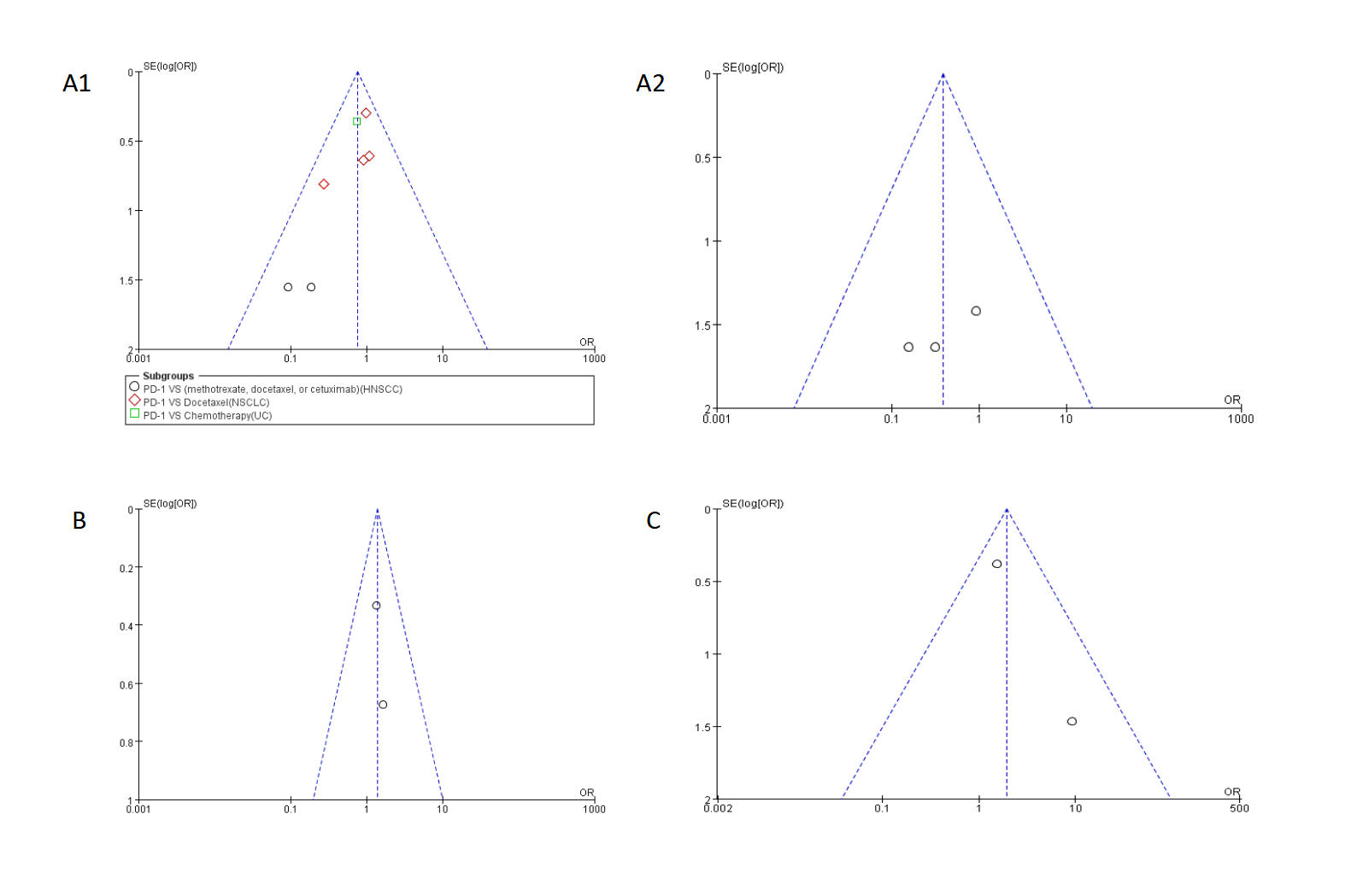

Supplement: Supplementary Figure 7 — Funnel plots of the risk of dizziness. (A1) The risk of all-grade dizziness calculated by the fixed effect (FE) model (PD-1/PD-L1 vs. chemotherapy): subgroup analysis was put into practice based on PD-1/PD-L1 and tumor types in both groups. (A2) The risk of dizziness of grades 3–5 calculated by the fixed effect (FE) model (PD-1/PD-L1 vs. chemotherapy). (B) The risk of all-grade dizziness calculated by the fixed effect (FE) model (PD-1/PD-L1 + chemotherapy vs. chemotherapy). (C) The risk of all-grade dizziness calculated by the fixed effect (FE) model (PD-1/PD-L1 + CTLA-4 vs. CTLA-4). [file Image_7.tif]

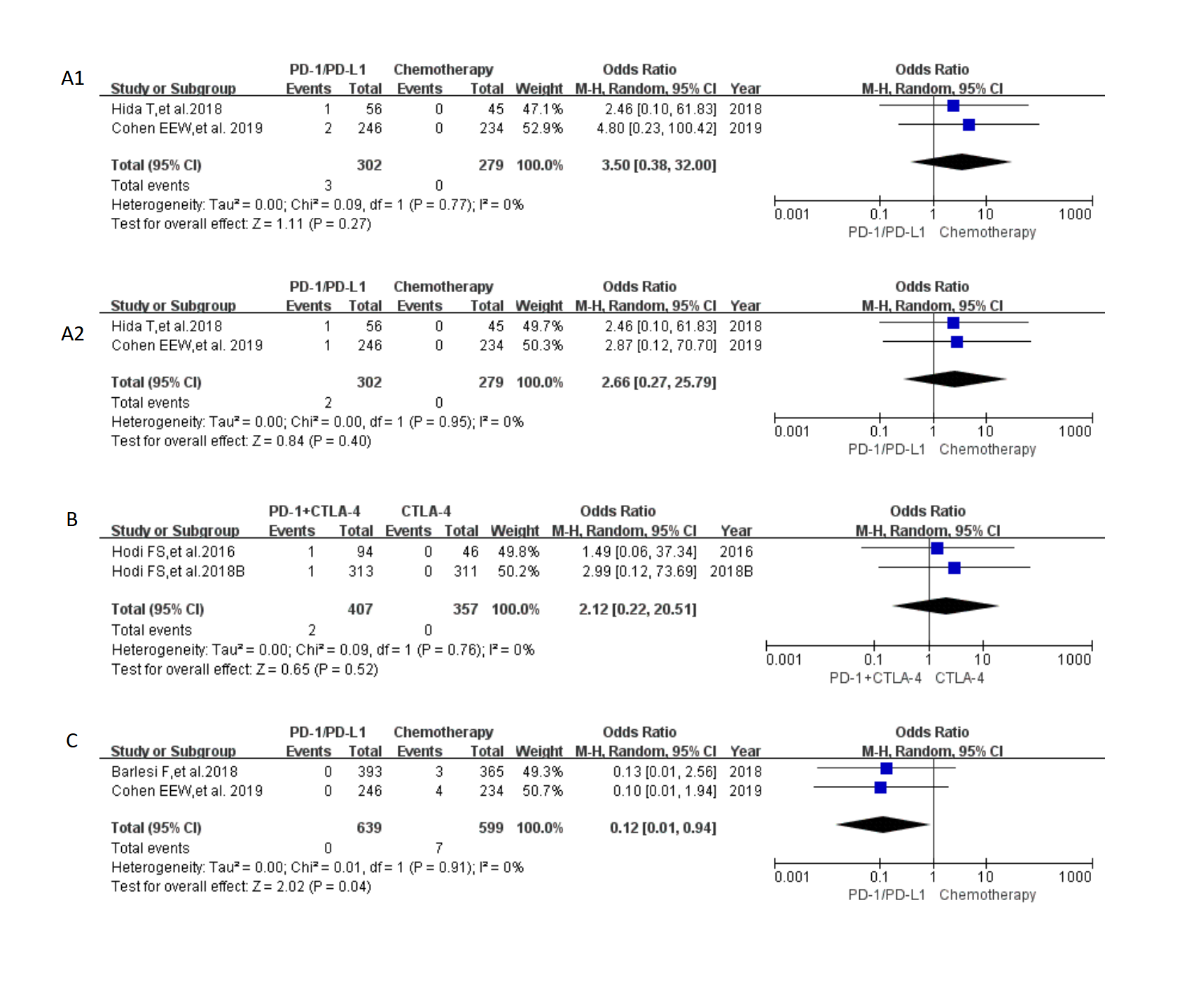

Supplement: Supplementary Figure 8 — Forest plots of the risk of rarely reported neurological toxicities. (A1) The risk of all-grade Guillain–Barré Syndrome calculated by the random effect (RE) model (PD-1/PD-L1 vs. chemotherapy): subgroup analysis was put into practice based on PD-1/PD-L1 and tumor types in both groups. (A2) The risk of Guillain–Barré Syndrome of grades 3–5 calculated by the random effect (RE) model (PD-1/PD-L1 vs. chemotherapy). (B) The risk of all-grade Guillain–Barré Syndrome calculated by the random effect (RE) model (PD-1/PD-L1 + CTLA-4 vs. CTLA-4). (C) The risk of all-grade polyneuropathy calculated by the random effect (RE) model (PD-1/PD-L1 + CTLA-4 vs. CTLA-4). [file Image_8.tif]

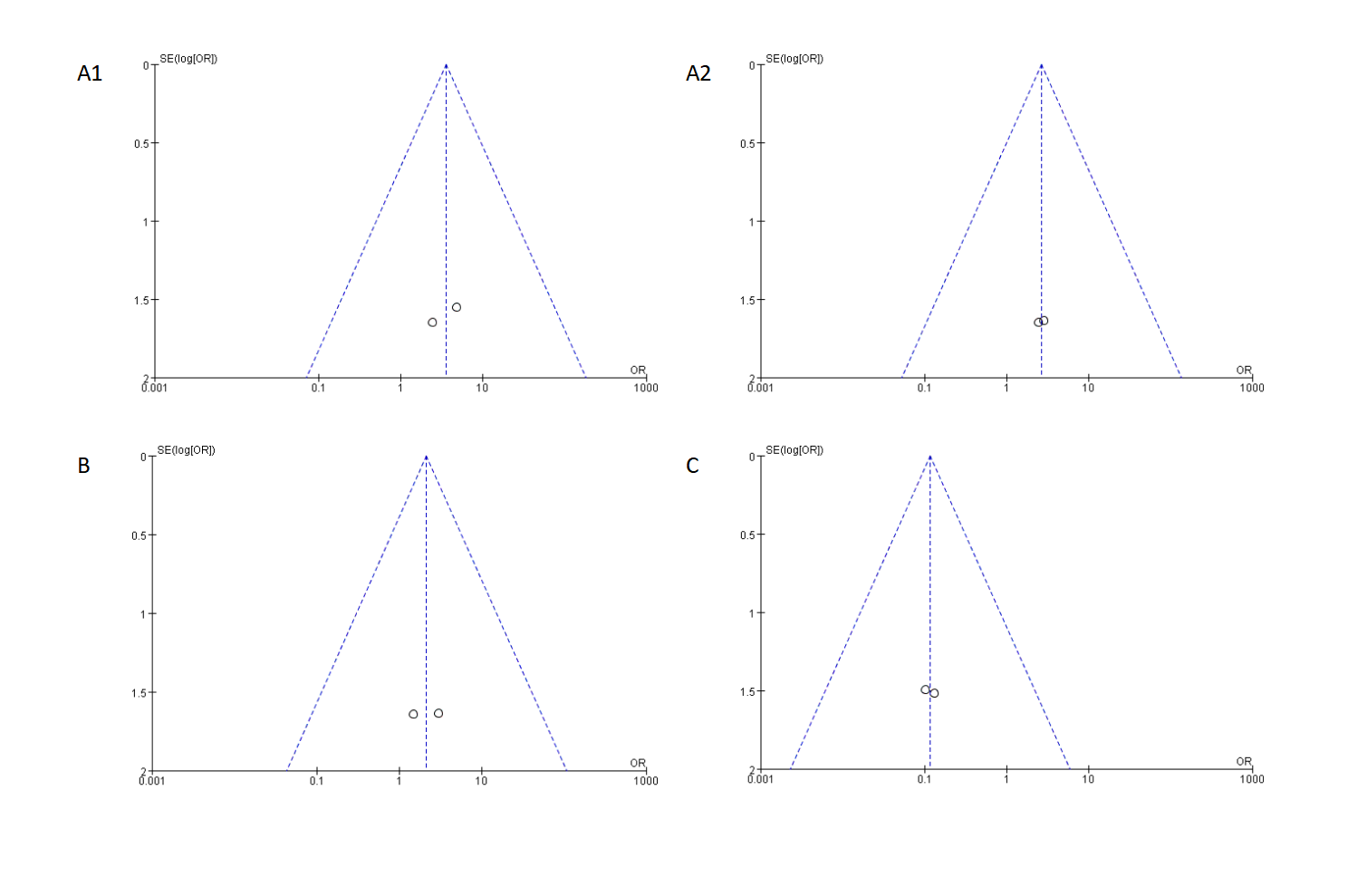

Supplement: Supplementary Figure 9 — Funnel plots of the risk of rarely reported neurological toxicities. (A1) The risk of all-grade Guillain–Barré Syndrome calculated by the fixed effect (FE) model (PD-1/PD-L1 vs. chemotherapy): subgroup analysis was put into practice based on PD-1/PD-L1 and tumor types in both groups. (A2) The risk of Guillain–Barré Syndrome of grades 3–5 calculated by the fixed effect (FE) model (PD-1/PD-L1 vs. chemotherapy). (B) The risk of all-grade Guillain–Barré Syndrome calculated by the fixed effect (FE) model (PD-1/PD-L1 + CTLA-4 vs. CTLA-4). (C) The risk of all-grade polyneuropathy calculated by the fixed effect (FE) model (PD-1/PD-L1 + CTLA-4 vs. CTLA-4). [file Image_9.tif]
